# Analysis of circulating microRNAs aberrantly expressed in alcohol-induced osteonecrosis of femoral head

**DOI:** 10.1038/s41598-019-55188-6

**Published:** 2019-12-12

**Authors:** Guoju Hong, Xiaorui Han, Wei He, Jiake Xu, Ping Sun, Yingshan Shen, Qiushi Wei, Zhenqiu Chen

**Affiliations:** 1 Devision of Orthopeadic Surgery, the University of Alberta, Edmonton, Alberta T6G 2R3 Canada; 2 The National Key Discipline and the Orthopedic Laboratory, Guangzhou University of Chinese Medicine, Guangzhou, Guangdong 510405 P.R. China; 3 School of Medicine, South China University of Technology, Guangzhou, Guangdong 510641 P.R. China; 4 Department of Orthopedic, the First Affiliated Hospital of Guangzhou University of Chinese Medicine, Guangzhou, Guangdong 510405 P.R. China; 5 Hip Preserving Ward, No. 3 Orthopaedic Region, the First Affiliated Hospital of Guangzhou University of Chinese Medicine, Guangzhou, Guangdong 510405 P.R. China; 6 School of Biomedical Sciences, the University of Western Australia, Perth, Western Australia 6009 Australia; 7 Department of Endocrinology, The First Affiliated Hospital of Guangdong Pharmaceutical University, Guangzhou, Guangdong 510080 P.R. China

**Keywords:** Metabolic disorders, Genetics research

## Abstract

Serum miRNAs are potential biomarkers for predicting the progress of bone diseases, but little is known about miRNAs in alcohol-induced osteonecrosis of femoral head (AIONFH). This study evaluated disease-prevention value of specific serum miRNA expression profiles in AIONFH. MiRNA PCR Panel was taken to explore specific miRNAs in serum of AIONFH cases. The top differentially miRNAs were further validated by RT-qPCR assay in serum and bone tissues of two independent cohorts. Their biofunction and target genes were predicted by bioinformatics databases. Target genes related with angiogenesis and osteogenesis were quantified by RT-qPCR in necrotic bone tissue. Our findings demonstrated that multiple miRNAs were evaluated to be differentially expressed with high dignostic values. MiR-127-3p, miR-628-3p, and miR-1 were downregulated, whereas miR-885-5p, miR-483-3p, and miR-483-5p were upregulated in serum and bone samples from the AIONFH patients compared to those from the normal control individuals (*p* < 0.01). The predicted target genes of the indicated miRNAs quantified by qRT-PCR, including *IGF2*, *PDGFA*, *RUNX2*, *PTEN*, and *VEGF*, were presumed to be altered in necrotic bone tissue of AIONFH patients. The presence of five altered miRNAs in AIONFH patients may serve as non-invasive biomarkers and potential therapeutic targets for the early diagnosis of AIONFH.

## Introduction

Alcohol-induced osteonecrosis of the femoral head (AIONFH), as defined by World Health Organization (WHO), is characterized by ischemia to the bone and bone marrow tissues within the constituents of the femoral head due to alcohol consumption^1^. AIONFH is an unpreventable and devastating disease. If left untreated, AIONFH can result in collapse of the femoral head that necessitates hip replacement in approximately 80% of patients^2,3^. While there are various theories regarding the causes of AIONFH, such as the extravascular accumulation of thrombosis mediating vascular constriction, the intravascular blood coagulation theory, the pathogenesis of AIONFH is still unclear^4,5^.

There is currently a lack of accurate and early diagnostic approach for ONFH, and only histological examination and magnetic resonance imaging (MRI) have been employed to date^6^. However, only if patients undergo hip arthroplasty in late stages of the disease (Association Research Circulation Osseous (ARCO) III and IV phase), histological examination can be utilized in the evaluation of the isolated femoral head. MRI enables to detect the AIONFH early to ARCO I phase, but the images captured in this phase are usually at low resolution and easy to be misdiagnosed by physicians. Investigation of microRNAs (miRNAs) in osteonecrosis of femoral head (ONFH) is beneficial to characterization of cellular cross-talk between endothelial cells and osteocytes as well as identification of detailed molecular mechanisms and biomarker regulation patterns. A handful of possible biomarkers for steroid-induced osteonecrosis of femoral head (SIONFH) have been reported^7–9^, suggesting that serum biomarkers may be also used to feasibly detect AIONFH in an earlier time point. Furthermore, the screening of biomarkers in AIONFH patients may contribute to the exploration of the microfluidics-based PCR used in AIONFH. However, specific biomarkers in AIONFH patients have not been well established. In this study, we focused on the investigation of specific miRNAs and aimed to identify potentially novel diagnostic biomarkers of AIONFH.

The interest in identifying miRNAs as diagnostic biomarkers in recent years has been focused on serum miRNAs^10,11^. An miRNA is a noncoding, short RNA segment approximately 22 nucleotides in length^12^. In bone microenvironment, miRNAs are considered to be involved in the proliferation, differentiation, and apoptosis of osteoclasts, osteoblasts and osteocytes^13^. They regulate the expression of mRNA in the posttranscription process by inducing the RNA silencing complex^14^. There are two main functions for the silencing mechanism: one results in mRNA target cleavage, and the other leads to mRNA degradation or repression of protein translation. In the differentially expressed miRNAs in SIONFH patients with systemic lupus erythematosus (SLE), 15 miRNAs have been found to be overexpressed while 12 to be downregulated^9^. Other experiments with various gene chips and microarrays have been performed in cells extracted from human tissues or animal model receiving steroid interventions and showed that miRNAs may also play a significant role in regulating bone cells and bone homeostasis^15–19^. However, the role of serum miRNAs is still unclear in human AIONFH patients.

AIONFH is an unpreventable musculoskeletal disease occurring in the femoral head that frequently requires total hip arthroplasty. Hence, it is imperative to identify noninvasive diagnostic and predictive biomarkers for AIONFH. In this study, we investigated whether specific miRNAs are differentially expressed in patients with AIONFH. Our results revealed miRNAs as novel potential biomarkers for the early diagnosis of AIONFH and might promote development of innovative treatment for AIONFH.

## Materials and Methods

### Subjects and samples

A total of forty AIONFH subjects were recruited from Department of Orthopedic, the First Affliated Hospital of Guangzhou University of Chinese Medicine in Guangzhou, China between June 2016 to Feburary 2017. AIONFH was diagnosed based on MRI evaluations and then classified according to the *Association Research Circulation Osseous* (ARCO) Stage system. Patients were invovled with the following criteria to reduce the unexpected impacts of biases: (a) with no interventions contraindicated, (b) age younger than 50 years; (c) presumptive long-term alcohol consumption; and (d) MRI and X-ray examinations revealing ARCO stage II to III disease duration. The exclusion criteria were as follows: (a) personal history of taking steroidal hormones; (b) ARCO stage IV. We also recruited the same number of age−/sex-matched volunteers as the control group. The inclusion criteria for the control group were as follows: (1) healthy individuals with no history of serious diseases or hormone use; (2) without ONFH; (3) suffered from traumatic femoral neck fracture or underwent total hip arthroplasty. All the study participants were of Chinese Han nationality with no blood relationship to one another. The detailed personal characteristics of the patient and control groups were profiled in Sup. Table 1. Participants involved in the study have been informed by details of study process and written informed consents were obtained from all participants. The study was conducted ethically in accordance with the World Medical Association Declaration of Helsinki and approved by the local ethical review committee of the First Affiliated Hospital of Guangzhou University of Chinese Medicine (no. ZYYECK[2017]069). The clinical trial was registered in a Chinese Clinical Trail Registry (ChiCTR) (No. ChiCTR-RPC-15006290).

### RNA extraction and miRNA expression profiles

Total miRNAs were measured from serum consisting of five AIONFH serum samples and five healthy serum samples to identify differentially expressed miRNAs. miRNAs were isolated and extracted from serum in a standard method according to the manufacturers’ recommendations (Invitrogen, Carlsbad, CA, USA). Briefly, the serum samples from both groups were centrifuged at 13000 × g for 5 min at 4 °C to eliminate pellet and cell debris. The supernatant serum was collected and added to TRIzol-LS (Invitrogen, Carlsbad, CA, USA), and then high-speed centrifugation was performed to obtain the aqueous phase. To precipitate the total RNA, serum was mixed with isopropyl alcohol and subjected to one further high-speed centrifugation at 10000 × g for 5 min at 4 °C. The total RNA was eluted in RNase-free water and stored at −80 °C until analysis. The amount and purity of RNA was estimated by ultraviolet spectrophotometer.

MiRNAs were profiled with miRCURY LNA^TM^ Universal RT microRNA PCR Panels (Exiqon, Vedbaek, Denmark) as described^20^. Briefly, RNA samples were diluted to 1.5–1.8 ng/μl using nuclease-free water. The specific miRNA primers for reverse transcription (RT) were used to amplify miRNAs in an RT reaction mix (Exiqon, Denmark) and subjected to amplification using SYBR™ Green Master Mix (Exiqon, Denmark) with an ABI PRISM 7900 Real-time PCR System (Applied Biosystems, USA). Quantitative miRNA expression levels were analyzed by 2^−ΔCt/ΔCt^ calculation with GenEx qPCR analysis software (Exiqon, Vedbaek, Denmark). Fold changes >2-fold or *p* < 0.05 were used to identify miRNAs that had significantly altered expression between the patient group and the healthy control group.

### miRNA validation by Real-time PCR

To further validate the miRNA array data, the differentially expressed miRNAs were analyzed with quantitative RT-PCR (qRT-PCR) in duplicate using an miRNA assay kit (GenePharma, Shanghai, China) in 30 serum samples (15 AIONFH patients and 15 healthy controls with femoral neck fracture) and 30 bone samples (15 AIONFH patients and 15 healthy controls with femoral neck fracture) as described^21,22^. Serum and femoral head isolated or detached from patients and volunteers were stored in −80 °C. Necrotic bone tissue was partially evaluated by histological examination and tissue with empty lacuna rate more than 50% was identified as necrotic area (Sup. Fig. 1). To isolate miRNA from serum and bone tissue, extraction with TriReagent (Invitrogen life technologies) was performed after milling of the tissue using liquid nitrogen.

The RT reaction was performed under the following reaction conditions: 30 min at 25 °C, 30 min at 42 °C, and 5 min at 85 °C, followed by maintenance at 4 °C. The selected miRNAs were confirmed with SYBR Green I dye (Takara, Dalian, China) with an ABI PRISM 7300 Real-time PCR System (Applied Biosystems, USA) at 95 °C for 3 min, followed by 40 cycles at 95 °C for 12 s and 62 °C for 40 s. Cel-miRNA-39-3p, a nonhuman miRNA, was spiked into the RNA samples as a control for the extraction and amplification steps. GAPDH was used for normalization of serum samples following the miRNA PCR array analysis.

Furthermore, receiver operating characteristic (ROC) curves were used to determine the diagnostic potential of serum miRNAs, which were generated after logarithmic transformation of all the samples included in the validation.

### Bioinformatics analysis of miRNA data

To estimate the potential biofunction and signaling pathways as well as target genes of miRNAs validated by real-time PCR, several bioinformatics analysis technologies were employed. Known putative targets of the selected miRNAs were generated by total three gene databases: TargetScan (http://www.targetscan.org/), miRanda (http://www.microrna.org/microrna/home.do), and MiRDB (http://www.mirbase.org). The predicted target genes from the three gene databases were summarized and ranked in order to obtain objective results with context scores and to minimize the false positive rates. Gene ontology (GO) analysis was utilized to identify the main biofunctions of the specific differentially expressed miRNAs, according to GO (http://www.geneontology.org/). The Kyoto Encyclopedia of Genes and Genomes (KEGG, http://www.genome.jp/kegg/) was used as a tool in our study to manage pathway analysis. All the involved data were considered based on their conserved sequences and the prospect of regulation of genes or pathways via miRNA activity.

### Quantitative real-time PCR of angiogenesis- and osteogenesis-related genes

The qRT-PCR system was used to quantified miRNAs related to mechanisms of osteogenesis and angiogenesis. These include insulin-like growth factor 2 (*IGF2*), semaphorin 3D (*SEMA3D*), runt-related transcription factor 2 (*RUNX2*), platelet-derived growth factor subunit A (*PDGFA*), superoxide dismutase 1 (*SOD1*), telomerase-associated protein 1 (*TEP1*), and vascular endothelial growth factor A (*VEGFA*). cDNA from ten necrotic bone tissue samples were used to compare with ten control bone tissue samples from femoral neck fracture patients. The relative expression levels of each gene were determined by qRT-PCR using the 2−ΔΔ CT method as described previously^23^, with β-actin as a normalized control. The sequences of both the forward and reverse primers of all genes involved are listed in Sup. Table 2.

### Statistical analyses

All statistical results are performed using SPSS (SPSS, IBM, USA) and GraphPad Prism (GraphPad Software, San Diego, CA, USA). Data was presented as the mean ± SD. Differences in the relative amount of miRNA between AIONFH patients and those in the control group were compared by using Student’s *t*-test or Welch’s *t*-test for equal or unequal variance. A two-tailed Mann-Whitney U test was utilized to compare different expression levels from samples from AIONFH patients compared with those from non-AIONFH patients. Fisher’s exact test and the *χ2* test were used to classify the GO category and select the significant pathway, and the false discovery rate (FDR) was used to correct the *p* value. We chose only GO terms with *p*-values < 0.01 and FDRs <0.01, and KEGG with *p*-values < 0.05 and FDRs <0.05. To identify the diagnostic sensitivity and specificity of serum miRNAs, ROC curves were used. The *p*-value tested the null hypothesis that the area under the curve was equal to 0.50. The cut-off points with the highest sensitivity and specificity were measured. The minimum level for significant differences was *p* < 0.05 or *p* < 0.01.

## Results

### miRNA expression patterns in the serum of AIONFH patients

**Figure 1:**
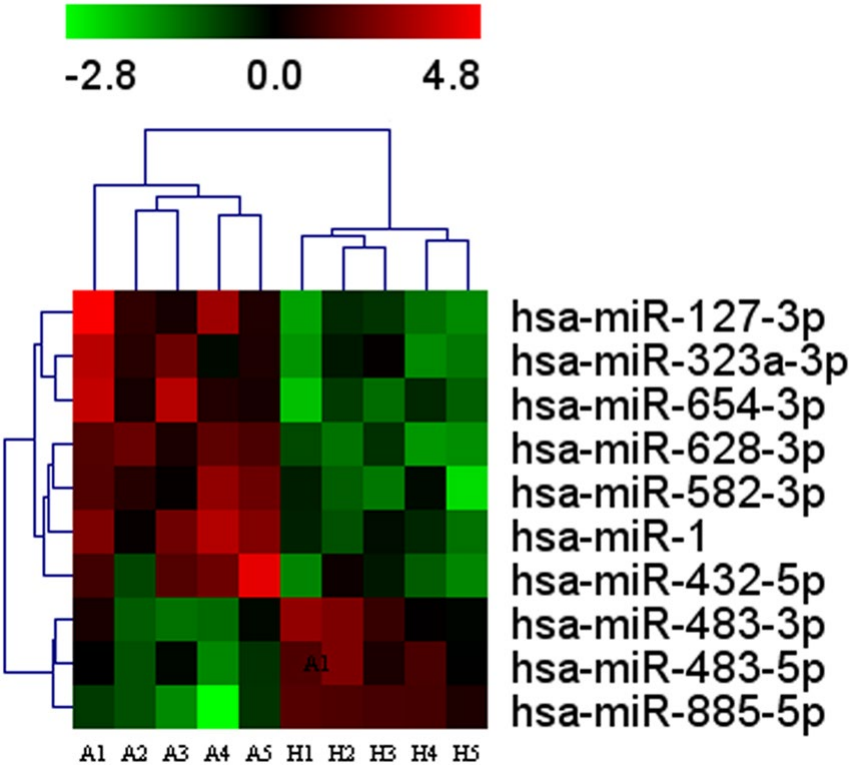
Differentially expressed miRNAs in serums of AIONFH patients by miRCURY LNATM Universal RT microRNA PCR Panels. A heatmap of differentially abundant miRNAs between AIONGH group and healthy control group (*p* < 0.01). (A: AIONFH patients, H: Healthy control; red and green bars indicated data of upregulated and downregulated miRNAs respectively).

**Figure 2:**
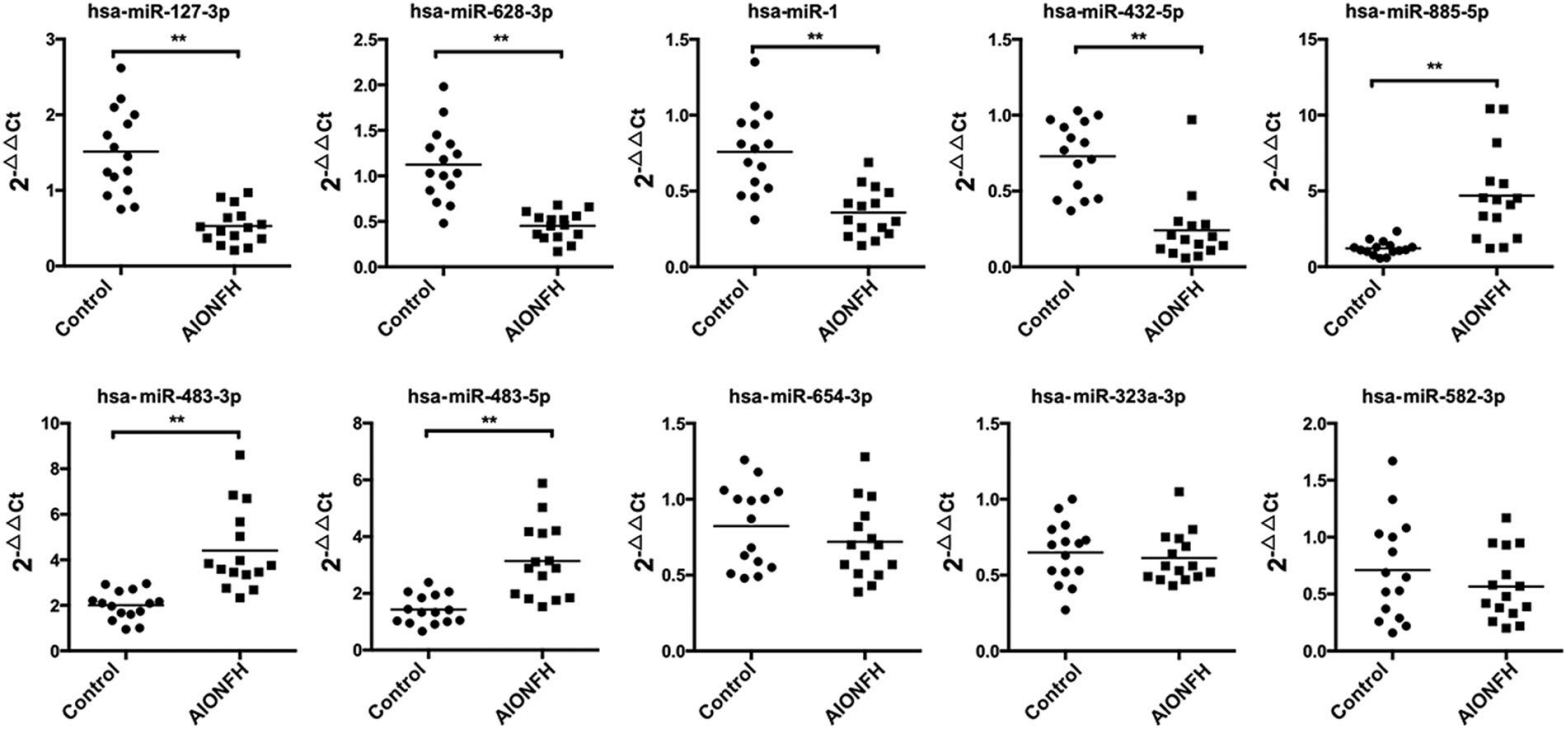
miRNAs are differently expressed in serum of AIONFH patients. Scatter plots provide the expression levels of specific miRNA in serum of AIONFH patients (n = 15) compared with health control group patients (n = 15). The expression of miR-127, miR-628-3p, miR-1, miR-432-5p, miR-885-5p, miR-483-5p, miR-483-3p were found to be significantly different (*p < 0.05, **p < 0.01 for the comparison indicated by Mann‐Whitney U test).

Initially microRNA PCR Panels were applied to detect the miRNAs spectra in serum samples from AIONFH patients and healthy control. The hierarchical clustering **(**Fig. 1**)** illustrated the differential miRNA expression profiles. Specifically, miR-127-3p, miR-1, miR-654-3p, miR-628-3p, miR-432-5p, miR-582-3p, and miR-323a-3p were significantly downregulated (*p* < 0.01), while miR-483-5p, miR-483-3p, and miR-885-5p were markedly upregulated (*p* < 0.01) **(**Table 1**)**. The qRT-PCR was performed to further validate the results, and our findings revealed that miR-127, miR-628-3p, miR-1, and miR-432-5p were downregulated (*p* < 0.05), miR-885-5p, miR-483-3p, and miR-483-5p were upregulated (*p* < 0.05). Interesting, miR-654-3p, miR-323a-3p, and miR-582-3p were remained unchanged **(**Fig. 2**)**. Taken together, the finding confirmed seven differentially expressed miRNAs. MiR‐127‐3p was the most abundant miRNA in the samples of AIONFH patients with a 8.31‐fold change compared with the healthy control. The sequences of both the forward and reverse primers of miRNAs are listed in Table 2.

**Table 1:** Regulated miRNAs in the serum of AIONFH Patients Compared With Non-AIONFH Patients Using the miRNA PCR Array.

miRNA ID	2^−ΔCt^	Regulation	Fold change	T test
has-miR-127-3p	0.004029	downregulated	−8.307335383	0.002123023
has-miR-323a-3p	0.002552	downregulated	−4.808848914	0.010531803
has-miR-654-3p	0.002141	downregulated	−6.331017573	0.009868429
has-miR-628-3p	0.00284	downregulated	−6.154356431	0.000469023
has-miR-582-3p	0.001006	downregulated	−4.93479379	0.036414245
has-miR-1	0.003439	downregulated	−6.51326985	0.001664897
has-miR-432-5p	0.002675	downregulated	−5.464401158	0.02435528
has-miR-483-3p	0.015391	upregulated	3.594748902	0.019106386
has-miR-483-5p	0.041276	upregulated	3.384004296	0.015444597
has-miR-885-5p	0.196931	upregulated	5.804806595	0.042959388

**Table 2:** Sequences of both the forward and reverse primers of miRNAs in RT-qPCR.

mRNA ID	Forward and reverse primers	bp
hsa-miR-127-3p	F:5′GGTCGGATCCGTCTGAGC3′ R: 5′GTGCGTGTCGTGGAGTCG3′	62
hsa-miR-323a-3p	F:5′GGCACATTACACGGTCG R:5′GTGCGTGTCGTGGAGTCG3′	63
hsa-miR-654-3p	F:5′GCCTATGTCTGCTGACCA3′ R:5′CAGTGCGTGTCGTGGA3′	65
hsa-miR-628-3p	F:5′GGGGTCTAGTAAGAGTGGC3′ R:5′TGCGTGTCGTGGAGTC3′	62
hsa-miR-582-3p	F:5′GGGGGTAACTGGTTGAACAA3′ R:5′GTGCGTGTCGTGGAGTCG3′	65
hsa-miR-1	F:5′ GGGGTGGAATGTAAAGAAGT R:5′CAGTGCGTGTCGTGGAGT3′	66
hsa-miR-432-5p	F:5′GGGTCTTGGAGTAGGTCATT3′ R:5′CAGTGCGTGTCGTGGAG3′	66
hsa-miR-483-3p	F:5′GGGGTCACTCCTCTCCTCC3′ R:5′GTGCGTGTCGTGGAGTCG3′	63
hsa-miR-483-5p	F:5′GGGTAAGACGGGAGGAAAGA3′ R:5′GTGCGTGTCGTGGAGTCG3′	64
hsa-miR-885-5p	F:5′GCCCTTCCATTACACTACCCT3′ R:5′GTGCGTGTCGTGGAGTCG3′	65

### miRNA expression patterns in the necrotic bone of AIONFH patients

**Figure 3:**
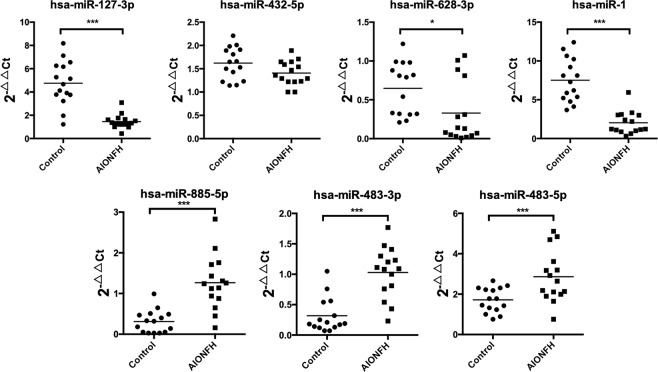
miRNAs are differently expressed in necrotic bone of AIONFH patients. Bar chat provide the expression levels of specific miRNA in necrotic bone of AIONFH patients (n = 15) compared with health control group patients (n = 15). The expression of miR-1, miR-127-3p, miR-483-5p, miR-483-3p, miR-628-3p, miR-885-5p were found to be significantly different (*p < 0.05, **p < 0.01, **p < 0.001 for the comparison indicated by Mann-Whitney U test).

We performed qRT-PCR in necrotic bone tissue samples to identify the expression of potential miRNAs. Our finding demonstrated that several indicated miRNA including miR-127, miR-628-3p, and miR-1 were downregulated (*p* < 0.05), while miR-885-5p, miR-483-3p, and miR-483-5p were markly upregulated (*p* < 0.05). The level of the distinguished miRNAs showed similar tendencies in bone as in serum. However, no change of miR-432-5p was observed in necrotic bone tissue samples **(**Fig. 3**)**. Therefore, our results revealed a close relationship between miRNAs and AIONFH and these six miRNAs were taken for the following analysis.

### Assessing the diagnostic value of miRNAs

**Figure 4:**
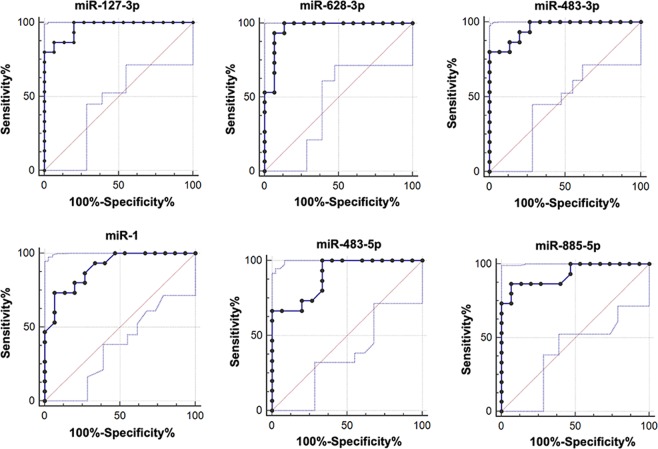
Diagnostic value of miRNAs for AIONFH. AUC for serum miRNAs: miR-1, miR-127-3p, miR-483-5p, miR-483-3p, miR-628-3p, miR-885-5p. The relative expressions of each miRNA were log‐transformed.

Serum miRNAs might have the potential to be used as early diagnostic biomarkers of AIONFH. Here we used ROC curve analysis to assess the relative expression of miRNAs after logarithmic transformation, which indicated their power for predicting the incidence of disease. The associated area under the curve (AUC) was used to confirm the diagnostic value of each seven miRNA identified in the AIONFH patients’ serum **(**Fig. 4**)**. The larger the area of AUC is, the higher its diagnostic value is. The AUC of miR-1 was the largest, reaching 0.904 (95% confidence interval (CI)^24^: 0.740–0.981, *p* < 0.0001). The AUC of the remaining six miRNAs was as follows: 0.960 for miR-483-3p (95% CI: 0.818–0.998, *p* < 0.0001), 0.900 for miR-483-5p (95% CI: 0.735–0.979, *p* < 0.0001), 0.964 for miR-628-3p (95% CI: 0.824–0.999, *p* < 0.0001), 0.931 for miR-885 (95% CI: 0.776–0.991, *p* < 0.0001), and 0.969 for miR-127 (95% CI: 0.831–0.999, *p* < 0.0001). The sensitivity and specificity associated with the optimal cut-off points were shown in Table 3.

**Table 3:** Sensitivity and Specificity of the Regulated miRNAs in the Serum of AIONFH and Non-AIONFH Patients.

miRNA ID	Sensitivity (%)	Specificity (%)
miR-127-3p	100.00	80.00
miR-1	73.33	93.33
miR-628-3p	93.33	93.33
miR-483-5p	100.00	66.67
miR-483-3p	80.00	100.00
miR-885-5p	86.67	93.33

### miRNA PCR array-based GO and pathway analysis

We explored the functions and potential signaling pathways of all the selected miRNAs with differential expression using the GO database and KEGG pathway analysis. Fisher’s exact test and the χ2 test were used to calculate *p-*values and FDRs. According to the standards of *p* < 0.05 (GO) and *p* < 0.05 (KEGG), significant functions and pathways were filtered. As shown in Sup. Fig. 2, the GO results demonstrated enrolled pathways related to signal transduction, cell differentiation, cell methylation, cell growth and apoptotic processes. The KEGG results showed that the Wnt signaling pathway and the PI3K-Akt and cancer pathways had the highest correlation with the selected miRNAs **(**Sup. Fig. 3**)**.

### Osteogenesis- and angiogenesis-related genes influenced by the identified miRNAs

**Figure 5:**
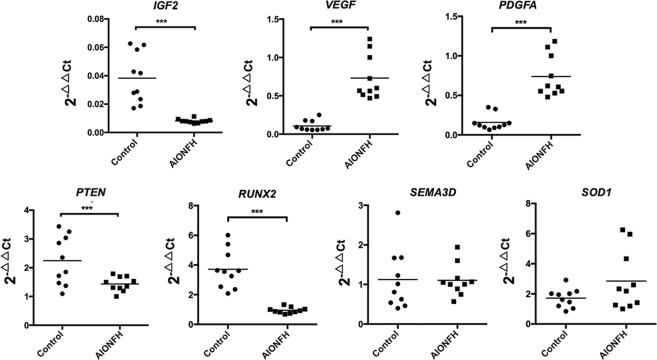
Potential genes modulated by specific miRNA in bone samples of AIONFH patients. Scatter plots reveals gene expression levels in bone tissue of AIONFH patients (n = 10) comparing with healthy control group (n = 10). It is demonstrated that *VEGF*, *PDGFA* were upregulated, whereas *RUNX2*, *PTEN*, *IGF2* was downregulated. *SOD1* and *SEMA3D* were no significant different between both groups. (*p < 0.05, **p < 0.01 for the comparison indicated by Mann-Whitney U test, *RUNX2* = runt-related transcription factor 2, *PTEN* = Phosphatase and Tensin Homolog, *VEGF* = Vascular Endothelial Growth Factor, *PDGFA* = Platelet Derived Growth Factor Subunit A, *IGF2* = Insulin Like Growth Factor 2, *SOD1* = Superoxide Dismutase 1, *SEMA3D* = Semaphorin 3D).

Sequencing analyses of the TargetScan, miRanda Microcosm and MiRDB databases demonstrated that several genes were predicted to be induced targets mediated by the potential miRNAs for both osteoblasts and osteoclasts **(**Sup. Table 3 and Sup. Fig. 4**)**. In addition, several potential genes related to angiogenesis and osteogenesis functions were found to be correlated with the identified miRNAs in the literature research (Pubmed, https://www.ncbi.nlm.nih.gov/pubmed/). To declare the correlation between indicated miRNAs and their predicted target genes, the mRNA transcript in necrotic bone samples from AIONFH patients was tested **(**Fig. 5**)**. In our report, *VEGFA* and *PDGFA* are predicted to be upregulated by miR-1 and *VEGFA* is modulated by miR-127-3p. By contrast, *PTEN* and *RUNX2* are predicted to be suppressed by miR-628-3p and *IGF2* is downregulated by miR-483-5p. However, the expression of *SEMA3D*, which is regulated by miR-885-5p, and *SOD1*, which is regulated by miR-1, are not significantly different in samples from AIONFH individuals.

## Discussion

In ONFH, interruption of vessels and an imbalance in osteoblast-osteoclast coupling causes alterations in bone mass and structure. miRNAs play a critical role in skeletal diseases such as osteonecrosis^6,19^. The diagnostic potential of measuring miRNAs in the serum of ONFH patients has been reported in patients with SIONFH. In particular, several miRNAs present in ONFH tissues and cells have been correlated with osteogenesis^18,19,25^. In the mesenchymal stem cells (MSCs) of patients with ONFH, *SMAD3* and *SMAD7* have been identified as the target genes of miR-708 and miR-17-5p, respectively, and their downstream signaling pathways are involved in osteogenic differentiation^7,25^. In hormone-induced rat models of osteonecrosis, miR-672-5p and miR-146a have been found to improve osteoblast formation^26^. Furthermore, some miRNAs involved in angiogenesis were also found to enhance the activity of angiogenic factors including VEGF, basic fibroblast growth factor (bFGF), tumor necrosis factor alpha (TNF-α) and proliferating cell nuclear antigen (PCNA)^8^, while some miRNAs, such as miR-34a, were found to be active in osteoblastic differentiation and endothelial coupling activity^27^.

To better understand the underlying mechanisms of AIONFH and to develop a potential early diagnostic biomarker for AIONFH, we investigated differentially expressed miRNAs in the serum of AIONFH patients with microarray analyses and identified seven specific miRNAs by RT-PCR. Among six of them, miR-127, miR-628-3p and miR-1 were downregulated, whereas miR-885-5p, miR-483-3p, and miR-483-5p were upregulated both in the serum and necrotic bone tissue samples from AIONFH patients. The expression level of each miRNA in the serum of AIONFH patients and controls was identified by relative concentration analysis with miR-432-5p, which is easy to yield better conclusion by comparing them with each other. In addition, we established the sensitivity and specificity with measurements of miRNAs using ROC curve to identify their diagnostic value in AIONFH.

All the above mentioned miRNAs identified in the serum of participants in our experiment, except for miR-885-5p, have effects on osteoblast, osteoclast and endothelial cell development for osteonecrosis healing. Induction of these particular miRNAs was highly effective in posttranscriptional control, which inhibited or enhanced the expression of various cytokines, thus allowing regulation of angiogenesis and osteogenesis in AIONFH. To establish the network of miRNAs posttranscriptional control and figure out their downstream genes, we combined the results of target genes prediction and literature review, and have quantitative measurements of those involved factors by using qRT-PCR.

As previously reported, overexpression miR-628-3p has a suppressive effect on osteogenesis and osteoblast differentiation via the downregulation of *RUNX2* mRNA or protein levels^28^. We found 0.4-fold significant downregulation of miR-628-3p in the serum and bone of AIONFH patients. The downregulation may lead to the suppression of *RUNX2* gene expression, resulting in the absence of more mature osteoblasts in AIONFH patients than in control individuals, which may explain the bone loss and microstructural disorders.

MiR-483-5p, which are both encoded within the intron of host *IGF2* gene, acts as suppressive factors^29,30^. In osteoporosis, only upregulation of miR-483-5p has a negative effect on *IGF2* and inhibits osteoblast differentiation by combining with the 5′-UTR of the fetal *IGF2* promoter transcript^31,32^. Our investigation in serum showed a strong upregulation of miR‐483-5p, and the PCR analysis showed that *IGF2* expression was suppressed in bone samples from AIONFH patients, which suggested that miR-483 was sufficient to predict the disease precisely and inhibit osteoblast in AIONFH through *IGF2*. It is also mentioned that upregulation of miR-483-5p and miR-483-3p have an negative effect on the migration endothelial progenitor cells and tube formation by targeting in serum response factors^29,30^. However, it is not consistent with the upregulated trend of *VEGF*. Further studies are required to clarified the competing results. No potential correlation with target genes was found in miR-483-3p.

MiR-127 contributes greatly to osteogenesis and angiogenesis in microenvironment. Specifically, the downregulated expression of miR-127 precursors has a potential effect on improving osteoblast mineralization and suppressing osteoclast differentiation in ovariectomized mice^33^. It is indicated that miR-127 is greatly downregulated in our study. Hence, we hypothesized that miR-127 may act as a reactive and positive role in AIONFH even osteoblast differentiation was suppressed in the femoral head. One analysis of ionizing radiation incidentally revealed a correlation between downregulated miR-127 and increased sensitivity of endothelial cells^34^. Due to a positive effect on angiogenic factors miR-127 hold, we considered the upregulated expression of *VEGF* is correlated to the identified miR-127 in AIONFH patients. This may lead to compensatory increases in vascularization in necrotic bone tissues.

The function of miR-1 has been comprehensively investigated in multiple previous reports. The critical role of miR-1 in angiogenesis was first identified in cardiovascular repair^35^. As mentioned, miR-1 is highly expressed in cardiomyocytes and cardiomyogenesis^36,37^. Lu also confirmed the specific function of miR-1 in cardiomyocytes and identified human frizzled-7 (*FZD7*) and fibroblast growth factor receptor substrate 2 (*FRS2*) as direct targets of miR-1^38^. In addition, miR-1 increased the expression levels of *VEGFA* in muscle in a zebrafish model and induced angiogenic signaling in the endothelium^39^, while the opposite results were found in osteosarcoma cells and gastric cells^40,41^. Furthermore, enhancer of zeste homolog 2 (*EZH2*) can suppress miR-1 transcription and promote angiogenesis^42^. In contrast, other reports have found that miR-1 increases the proliferation and migration or invasion of tumor endothelial cells^43,44^. During osteogenesis, miR-1 has been found to be related to *VEGFA*, *FGF2*, etc^45^. Interestingly, we detected lower expression levels of miR-1 and higher expression of *VEGF* in bone samples from AIONFH patients than in those from the control participants. However, regarding to bone metabolism there was still no direct evidence proving the correlation between *PDGFA* and miR-1 in literature study, even it was predicted in GO and KEGG analysis.

Taken together, our research evaluated serum miRNAs in AIONFH patients and investigated seven miRNAs that were differentially expressed in serum samples from these patients. In necrotic bone tissue, six differentially expressed miRNAs were identified. Three of these were concurrently downregulated in serum while the others were upregulated. These several significantly different circulating miRNAs might serve as novel biomarkers for the early diagnosis of AIONFH, as serum collection is convenient and noninvasive. Furthermore, our findings might provide potential novel targets for the pharmacological treatment of AIONFH in the future.

## Supplementary information

Supplemental information

## Data Availability

The datasets during the current study are available in the Mendeley database: PCR screening of miRNA in AIONFH cases and health control: 10.17632/sd88yf37r2.1; MiRNA (Bone) validation of AIONFH cases by Real-time PCR: 10.17632/g93vrx8kcz.1; MiRNA (serum) validation of AIONFH cases by Real-time PCR: 10.17632/nc65brcw8d.1; Target genes of miRNA in AIONFH cases: 10.17632/k8f976fpt2.1.
