# Supplementary material for: Analysis of circulating microRNAs aberrantly expressed in alcohol-induced osteonecrosis of femoral head
Source: Sci Rep. 2019 Dec 12;9:18926. doi: 10.1038/s41598-019-55188-6 (PMC6908598; doi:10.1038/s41598-019-55188-6)
Supplement: Supplementary file 1 — Supplemental information [file 41598_2019_55188_MOESM1_ESM.docx]

**Analysis of circulating microRNAs aberrantly expressed in alcohol-induced osteonecrosis of the femoral head**

Guoju Hong MD^1,^^2#^, Xiaorui Han MD^3#^, Wei He MD^4,5^, Jiake Xu PhD^6^, Ping Sun PhD^7^, Yingshan Shen^2^, Qiushi Wei MD^4,5*^, Zhenqiu Chen MD^4,5*^

^1^ Department of Surgery, the University of Alberta, Edmonton, Alberta, Canada, T6G 2R3

^2^ The National Key Discipline and the Orthopedic Laboratory, Guangzhou University of Chinese Medicine, Guangzhou, Guangdong, P. R. China, 510405

^3^ School of Medicine, South China University of Technology, Guangzhou, Guangdong, P. R. China, 510641

^4^ Department of Orthopedic, the First Affiliated Hospital of Guangzhou University of Chinese Medicine, Guangzhou, Guangdong, P. R. China, 510405

^5^ Hip Preserving Ward, No. 3 Orthopaedic Region, the First Affiliated Hospital of Guangzhou University of Chinese Medicine, Guangzhou, Guangdong, P. R. China, 510405

^6^ School of Biomedical Sciences, The University of Western Australia, Perth, Western Australia, Australia, 6009

^7^ Department of Orthopedic, The First Affiliated Hospital of Guangdong Pharmaceutical University, Guangzhou, Guangdong, P. R. China, 510080

^#^These authors contributed equally to this work.

*Corresponding Author 1

Zhenqiu Chen

Department of Orthopedic

the First Affiliated Hospital of Guangzhou University of Chinese Medicine

16^th^ Airport Road, Baiyun District

Guangzhou, Guangdong, 510407, P. R. China

Tel: +8613662395019

Fax: +86-20-36591321

E-mail: gdstjeeson@foxmail.com

*Corresponding Author 2

Qiushi Wei

Hip Preserving Ward, No. 3 Orthopaedic Region

the First Affiliated Hospital of Guangzhou University of Chinese Medicine

16^th^ Airport Road, Baiyun District

Guangzhou, Guangdong, 510407, P. R. China

Tel: +8613602407269

Fax: +86-20-36591321

E-mail: [weiqshi@126.com](mailto:weiqshi@126.com)

**
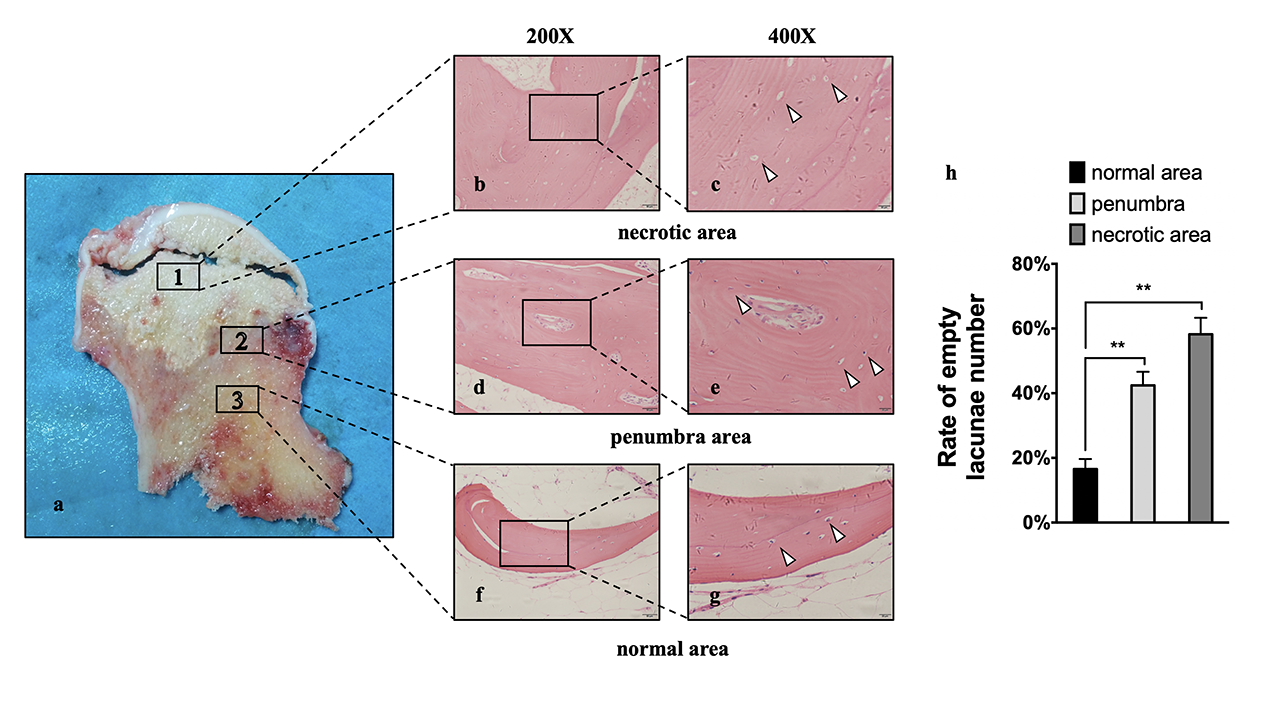
**

**Supplemental figure 1**


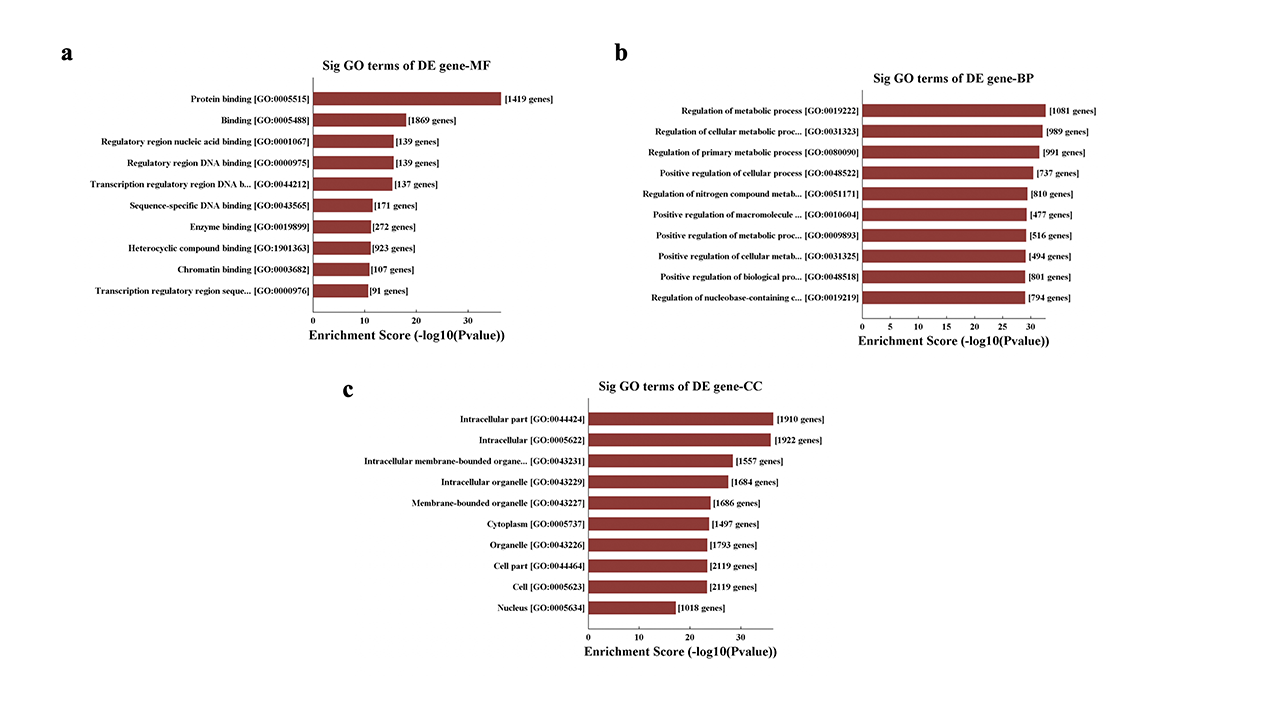


**Supplemental figure 2**


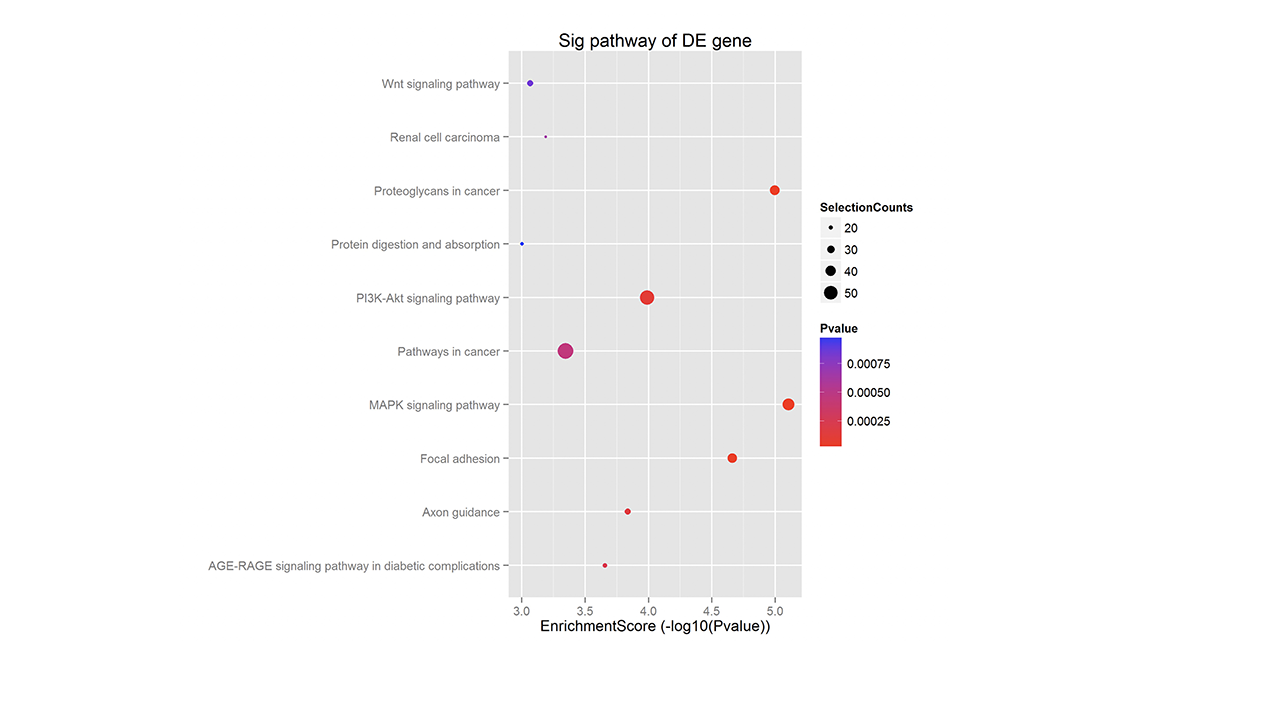
 **Supplemental figure 3**


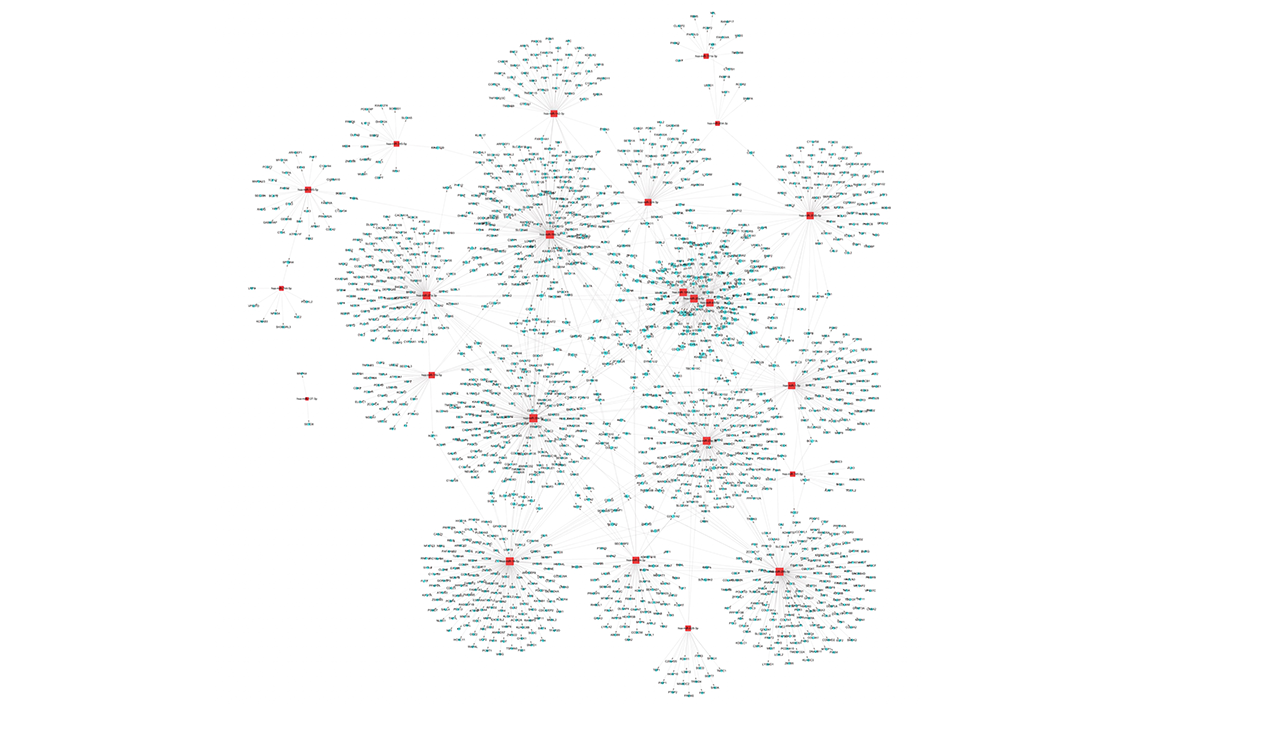
 **Supplemental figure 4**

**Supplemental figure legends**

Sup Fig. 1. Histologcial evaluation of femoral head isolated from AIONFH cases. (a) gross specimen slice of femoral head with distinguishable pathological areas as necrotic area (1), penumbra are (2) and normal area (3); Histopathological examination was performed in (b, c) normal area, (d, e) penumbra area and (f, g) focal area. Images were shown as ×200 and ×400. (h) Quantitative analysis of empty bone lacunae rate in the femoral heads of AIONFH patients.

Sup Fig. 2. Functional predictions of differentially abundant miRNAs in serums of AIONFH and healthy control group. The number of genes in GO term was shown in histograph. a-c, enriched biological processes, cellular components and molecular functions in ONFH were shown.

Sup Fig. 3. Scatterplot of enriched KEGG pathway showing statistics of pathway enrichment in AIONFH.

Sup Fig. 4. Network of target genes related with miRNAs.

**Supplemental Table 1**. Characteristics of AIONFH and non-AIONFH evolved in the research

| Patients characteristics | miRNA PCR array and RT-qPCR (Serum) | | RT-qPCR (Bone) | |
| --- | --- | --- | --- | --- |
|  | AIONFH group | Non-AIONFH group | AIONFH group | Non-AIONFH group |
| Number of hips | 20 | 20 | 10 | 10 |
| Age (years) | 37.3±8.2 | 35.1±9.2 | 36.4±11.2 | 37.3±7.7 |
| Males: females (no.) | 21:9 | 17:18 | 8:2 | 6:4 |
| Weight (kg) | 60.8±8.2 | 57.3±4.9 | 56.4±8.5 | 59.3±9.5 |
| ARCO Stage |  | | | |
| I stage | 0 | / | 0 | / |
| II stage | 29 | / | 0 | / |
| III stage | 6 | / | 3 | / |
| IV stage | 0 | / | 7 | / |

**Supplemental Table 2**. Sequences of both the forward and reverse primers of all target mRNA in RT-qPCR.

| mRNA ID | Forward and reverse primers | bp |
| --- | --- | --- |
| *β-actin* | F:5' GTGGCCGAGGACTTTGATTG 3'  R :5’ CCTGTAACAACGCATCTCATATT 3’ | 73 |
| *SEMA3D* | F:5' AAGGCTCTGCTGTTTGTG 3'  R :5’ CTTAATCAGTGGGTCATAGGT 3’ | 167 |
| *PDGFA* | F:5' CCAGGACGGTCATTTACG 3'  R :5’ GCAGCGTTTCACCTCCAC 3’ | 95 |
| *SOD1* | F:5' AAAGATGGTGTGGCCGATGT 3'  R :5’ CAAGCCAAACGACTTCCAGC 3’ | 167 |
| *TEP1* | F:5' GTTTACCTGTTGGACCTGA3'  R:5’AAGGCAGTAAGAAAGAGTGTAT 3’ | 113 |
| *RUNX2* | F:5' AGTGGACGAGGCAAGAGTTTC 3'  R :5’ GCGGGACACCTACTCTCATACT 3’ | 217 |
| *IGF2* | F:5' CGTGCTGCATTGCTGCTTAC 3'  R:5’TAGCACAGTACGTCTCCAGGAGG 3’ | 198 |
| *VEGF* | F:5' CATGCAGATTATGCGGATCAA 3'  R :5’ GCATTCACATTTGTTGTGCTGTAG 3’ | 82 |

**Supplemental Table 3**, Possible target gene of selected miRNAs predicted by databases and their related cellular functions

| miRNA ID | Possible target gene | Cell related |
| --- | --- | --- |
| miR-885-5p | *ADD1* | Osteoblast |
|  | *AHNAK* | Osteoblast |
|  | *ATF7* | Osteoclast |
|  | *FAM108C1* | Osteoclast |
|  | *SEMA3D* | Osteoblast and Osteoclast |
|  | *ZNF281* | Osteoblast |
| miR-1-3p | *CLCN3* | Osteoblast |
|  | *DDX5* | Osteoblast |
|  | *EIF4E* | Osteoblast |
|  | *FN1* | Osteoblast |
|  | *HSP90B1* | Osteoblast |
|  | *MAB21L1* | Osteoblast |
|  | *MMP8* | Osteoblast |
|  | *PDGFA* | Osteoclast and Osteoblast |
|  | *PTPRS* | Osteoblast |
|  | *SOD1* | Osteoclast and Osteoblast |
|  | *TMEM178* | Osteoclast |
| miR-628-3p | *ITPR3* | Osteoclast |
|  | *SLC40A1* | Osteoclast |
|  | *TEP1* | Osteoclast and Osteoblast |
| miR-432-5p | *TLN1* | Osteoclast |
| miR-483-3p | *ARRB2* | Osteoblast |
|  | *DLC1* | Osteoclast |
|  | *TEAD2* | Osteoblast |
| miR-432-5p | *TLN1* | Osteoclast |
| miR-127-3p | *SETD8* | Osteoclast |
